# Widely Targeted Metabolomics Reveal the Distribution of Metabolites in Shatian Pomelo Fruit

**DOI:** 10.3390/foods13223698

**Published:** 2024-11-20

**Authors:** Jing Wen, Haocheng Liu, Huining Lai, Yujuan Xu, Jijun Wu, Yuanshan Yu, Wenqian Huang, Manqin Fu, Haiyang Liu

**Affiliations:** 1 School of Chemistry and Chemical Engineering, South China University of Technology, Guangzhou 510641, China; jingw988@163.com (J.W.); ansishc@163.com (H.L.); 2 Institute of Sericulture and Agricultural Products Processing, Guangdong Academy of Agricultural Sciences/Key Laboratory of Functional Foods, Ministry of Agriculture and Rural Affairs/Guangdong Key Laboratory of Agricultural Products Processing, Guangzhou 510610, China; laihn2022@126.com (H.L.); xyj6510@126.com (Y.X.); wujijun@gdaas.cn (J.W.); yuyuanshan@gdaas.cn (Y.Y.); huangwenqian94@126.com (W.H.); fumanqin84@126.com (M.F.)

**Keywords:** Shatian pomelo, metabolomics, potential value, multi-tissue site

## Abstract

Using ultra-performance liquid chromatography-tandem mass spectrometry (UPLC-MS/MS) technology in multiple reaction monitoring mode, a widely targeted metabolomics approach was employed to identify metabolites in five tissues (exocarp, endocarp, segment membrane, pulp, and seeds) of the Shatian pomelo fruit. The differences in metabolite composition and abundance among different tissues were analyzed using multivariate statistical analysis methods. The results showed that a total of 1722 metabolites were identified from the five tissues of the Shatian pomelo, including 413 flavonoids and 277 amino acids and their derivatives. Flavonoid metabolites accumulate the most abundantly in the exocarp and seeds, while amino acids and their derivatives are primarily accumulated in the exocarp and pulp. A total of 649 key differential metabolites were screened, including flavonoids, amino acids, and their derivatives, indicating the presence of tissue-specific accumulation of metabolites in the Shatian pomelo. This study systematically investigated the metabolite distribution in different tissue parts of the Shatian pomelo, and validated the feasibility of widely targeted metabolomics technology in pomelo quality analysis. It provided a theoretical reference for metabolic research on the Shatian pomelo and other citrus fruits, and offered a theoretical basis for the efficient utilization of pomelo resources.

## 1. Introduction

Pomelo (*Citrus paradisi*), belonging to the genus Citrus in the family rutaceous, is a commercially important fruit that is widely grown worldwide [1]. In China, pomelo cultivation has a long history and is mainly distributed in the southern region. Pomelo is not only consumed as a fresh fruit but has also been utilized in the production of juices, beverages, canned goods, preserves, and other products [2,3,4,5]. Its rich medicinal value has been documented in ancient texts throughout the ages [6,7], highlighting the potential health benefits derived from its numerous bioactive compounds.

Numerous studies have focused on elucidating the nutritional and pharmacological properties of pomelo and its extracts. For instance, researchers have conducted experiments to demonstrate the antioxidant, anti-inflammatory, and anti-diabetic effects of pomelo extracts. In one study, the flavonoid-rich extract from pomelo peel was found to exhibit significant antioxidant activity, scavenging free radicals and protecting cells against oxidative stress [8]. Another experiment investigated the neuroprotective effects of pomelo seed extract, which contains high levels of the bioactive compound citrulline. It was demonstrated that citrulline inhibits Aβ25-35-induced neurotoxicity by activating the PI3K/Akt signaling pathway, inhibiting caspase-3, and upregulating Bcl-2 expression [9].

Moreover, studies have shown that pomelo peel extract, rich in flavonoids such as naringenin, naringin, and isonaringin, exerts a protective effect on neuroblastoma cell lines at appropriate concentrations, suggesting potential anticancer properties [10,11]. These findings underscore the potential therapeutic applications of pomelo extracts in the prevention and treatment of various diseases.

Ultra-performance liquid chromatography-tandem mass spectrometry (UPLC-MS/MS), as a widely targeted metabolomics technology, has emerged as a powerful tool for qualitative and quantitative analysis of metabolites in biological samples [12,13]. This technology combines the advantages of both targeted and non-targeted metabolomics, enabling rapid and accurate identification of thousands of metabolites through professional compound analysis software databases [14,15]. Previous studies utilizing UPLC-MS/MS have revealed significant metabolic pathway regulation during fruit processing and development, providing insights into the metabolic mechanisms underlying the accumulation of bioactive compounds [16,17].

Currently, while there have been several studies focusing on the nutritional and pharmacological properties of the pomelo, there is a lack of comprehensive and systematic investigations into the metabolite distribution across different tissue parts of the Shatian pomelo. Therefore, this study systematically investigated the metabolite distribution characteristics of different tissue parts of Shatian pomelo fruits (exocarp, endocarp, segment membrane, pulp, and seeds) using widely targeted metabolomics technology. The aim was to determine the differential distribution of metabolites in each part of the fruit and to provide a theoretical basis for the high-value comprehensive utilization of Shatian pomelo resources.

## 2. Materials and Methods

### 2.1. Materials Preparation and Extraction

Shatian pomelo, harvested from the cultivation base of Shatian pomelo in Meizhou, Guangdong Province, was mature and disease-free. Ten whole fruits were randomly selected, and appropriate amounts of samples were collected from five parts of each pomelo, namely, exocarp (**A**), endocarp (**B**), segment membrane (**C**), pulp (**D**), and seeds (**E**).

Samples were dried by a freeze dryer (Scientz-100F), and ground to powder by a grinder (MM 400, Retsch; 30 Hz, 1.5 min). We accurately weighed 50.00 mg of sample powder and added 1200.00 μL of −20 °C pre-cooled 70% methanol aqueous solution. During sample extraction, the samples were vortexed every 30 min for 30 s each time for a total of 6 times. The samples were centrifuged (9000× *g* for 3 min), the supernatant was taken, the samples were filtered using a 0.22 μm microporous filter membrane, and the filtered samples were transferred to the injection vials for UPLC-MS/MS (ABSciex QTRAP 6500, SCIEX, Boston, MA, USA) analysis.

### 2.2. UPLC-MS/MS Conditions

UPLC conditions: chromatographic column Agilent SB-C18 (1.8 μm, 2.1 mm × 100 mm); mobile phases: ultrapure water (phase A, containing 0.1% formic acid) and acetonitrile (phase B, containing 0.1% formic acid); gradient elution: 0.00 min A-phase proportion of 95%, B-phase proportion of 5%, within 9.00 min the proportion of A-phase linearly decreased to 5%, the B-phase proportion linearly increased to 95% in 9.00 min and maintained for 1 min, 10.00–11.10 min, the proportion of phase A increased to 95%, the proportion of phase B decreased to 5% and equilibrated to 14 min; flow rate of 0.35 mL/min, column temperature of 40 °C, injection volume of 2.00 μL.

ESI-Q TRAP-MS/MS: A triple quadrupole linear ion trap mass spectrometer (Q TRAP), the AB4500 Q TRAP UPLC/MS/MS system was equipped with an ESI Turbo ion spray interface, which can be run in both positive and negative ion modes using Analyst 1.6.3 software (AB Sciex, Framingham, MA, USA). The ESI source operation parameters were as follows: source temperature 500 °C; ion spray voltage (IS) 5500 V (positive ion mode)/−4500 V (negative ion mode); ion source gas I (GSI), gas II (GSII), curtain gas (CUR) was set at 50, 60, and 25 psi, respectively; the collision-activated dissociation (CAD) was high. QQQ scans were acquired as MRM experiments with collision gas (nitrogen) set to medium. DP (declustering potential) and CE (collision energy) for individual MRM transitions was done with further DP and CE optimization. A specific set of MRM transitions were monitored for each period according to the metabolites eluted within this period.

### 2.3. Quality Control (QC) Sample Determination

QC samples were prepared by mixing equal amounts of extracts (from Section 2.1) from 5 different parts of the Shatian pomelo samples in 3 replicates, which were processed and assayed in the same way as the analyzed samples, and 1 QC sample was inserted into every 10 assayed analyzed samples during the instrumental testing to examine the reproducibility of the whole analytical process.

### 2.4. Data Analysis

The metabolite data were all based on the Metware Wuhan Myway Biotechnology Co., Ltd., (Wuhan, China) MVDB V2.0 database and metabolite information in public databases. Primary and secondary mass spectrometry analysis is based on existing mass spectrometry databases such as MassBank, KNAPSAcK, HMDB, and METLIN. After obtaining the mass spectrometry data of metabolites in different samples, the software Analyst 1.6.3 was used to integrate the peak areas of all mass spectrometry peaks and integrate and correct the mass spectrometry peaks of the same metabolites in different samples. Peak areas were normalized to assess the relative content of metabolites, and heatmap analysis was performed using the OmicStudio tool (https://www.omicstudio.cn, accessed on 13 May 2024).

Unsupervised PCA (principal component analysis) was performed by the statistics function prcomp within R (www.r-project.org, accessed on 18 May 2024). The data were unit variance scaled before unsupervised PCA.

The HCA (hierarchical cluster analysis) results of samples and metabolites were presented as heatmaps with dendrograms, while Pearson correlation coefficients (PCC) between samples were calculated by the cor function in R and presented as only heatmaps. Both HCA and PCC were carried out by the R package ComplexHeatmap. For HCA, normalized signal intensities of metabolites (unit variance scaling) are visualized as a color spectrum.

For two-group analysis, differential metabolites were determined by VIP (VIP > 1) and absolute Log_2_FC (|Log_2_FC| ≥ 1.0). VIP values were extracted from the OPLS-DA results, which also contain score plots and permutation plots, and were generated using the R package MetaboAnalystR. The data were log transformed (log10) and mean centered before OPLS-DA. In order to avoid overfitting, a permutation test (200 permutations) was performed.

Annotations were made based on the retention time, mass-to-charge ratio, and peak intensity obtained. Identified metabolites were annotated using the KEGG Compound database (http://www.kegg.jp/kegg/compound/, accessed on 1 June 2024); annotated metabolites were then mapped to the KEGG Pathway database (http://www.kegg.jp/kegg/pathway.html, accessed on 10 June 2024). Pathways with significantly regulated metabolites mapped to were then fed into MSEA (metabolite sets enrichment analysis), and their significance was determined by a hypergeometric test’s *p*-values.

## 3. Results

### 3.1. Quality Control and Statistical Analysis of Widely Targeted Metabolomic Samples from Different Parts of the Shatian Pomelo

**Figure 1:**
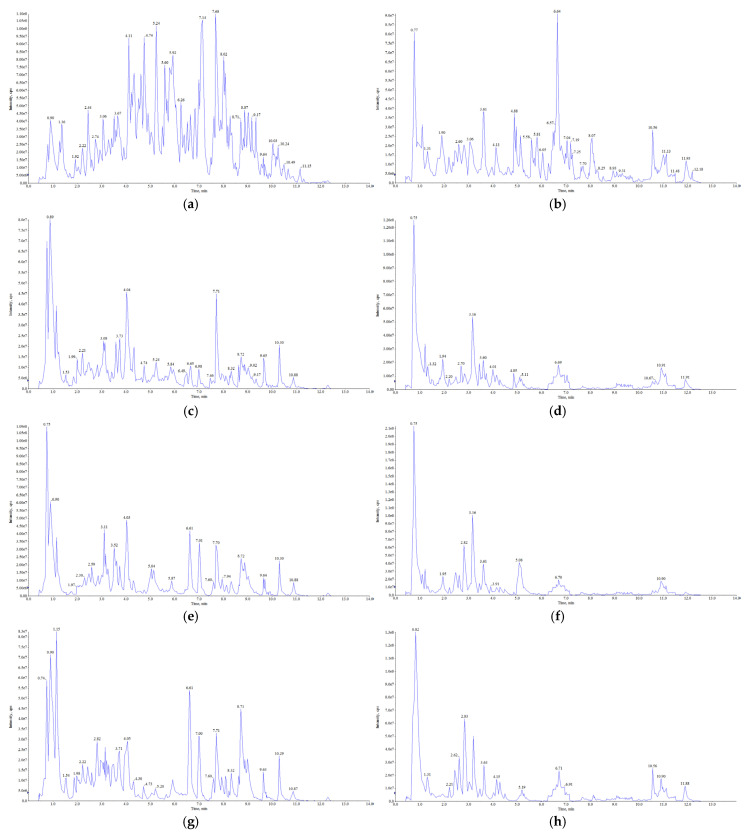

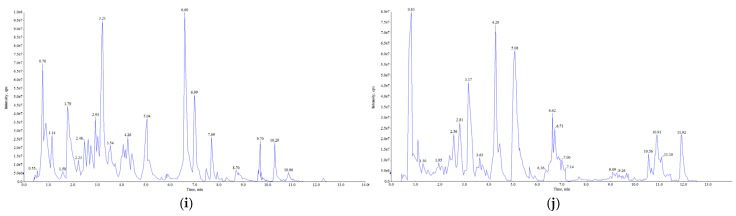
TIC plots of mass spectrometry of five parts of the Shatian pomelo ((**a**): positive ion mode of **A**—exocarp, (**b**): negative ion mode of **A**—exocarp, (**c**): positive ion mode of **B**—endocarp, (**d**): negative ion mode of **B**—endocarp, (**e**): positive ion mode of **C**—segment membrane, (**f**): negative ion mode of **C**—segment membrane, (**g**): positive ion mode of **D**—pulp, (**h**): negative ion mode of **D**—pulp, (**i**): positive ion mode of **E**—seed, (**j**): negative ion mode of **E**—seed).

The total ion current (TIC) plots of the QC samples showed the total intensity of all ions in the mass spectrometer at different time points (Figure 1a–j). The superposition of the TIC plots of the QC mass spectrometry showed a high overlap with the TIC curves of the metabolites, which illustrated the reproducibility and reliability of the data (Figure S1a–d). Multiple peak detection plots of metabolites in multiple reaction monitoring (MRM) mode showed ion current maps for multiple substances. In the MRM plots, each differently colored mass spectrometry peak represents a detected metabolite (Figure S1e,f).

**Figure 2:**
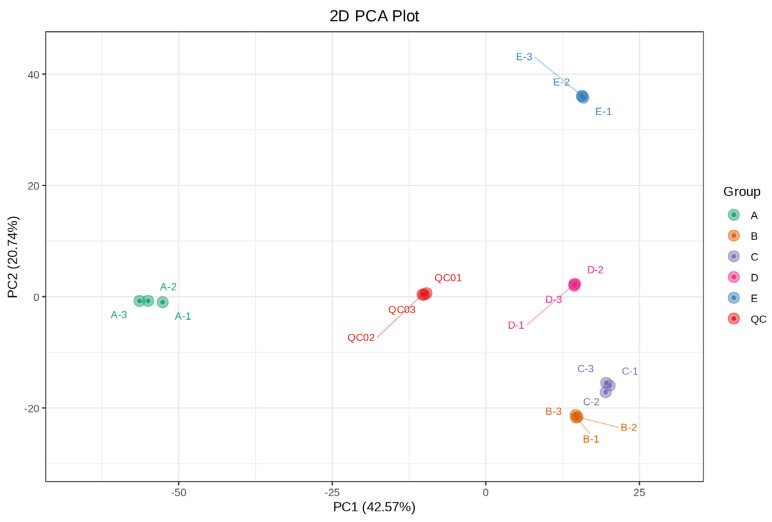
Principal component analysis (PCA) of metabolites and QC samples from five parts of the Shatian pomelo (**A**—exocarp, **B**—endocarp, **C**—segment membrane, **D**—pulp and **E**—seed).

Principal component analysis (PCA) was performed on all duplicates as well as QC samples in order to gain a preliminary understanding of the overall metabolite differences between groups of samples and the magnitude of variability between samples within a group in order to assess the quality of the metabolic profiling data. All samples in Figure 2 clustered significantly in PCA with low variability, suggesting that the data of each sample were reproducible and reliable for differential metabolite screening. Five parts of the Shatian pomelo were clearly separated from the QC samples, indicating that their metabolites differed significantly.

### 3.2. Composition and Classification of Metabolites from Different Parts of the Shatian Pomelo

**Figure 3:**
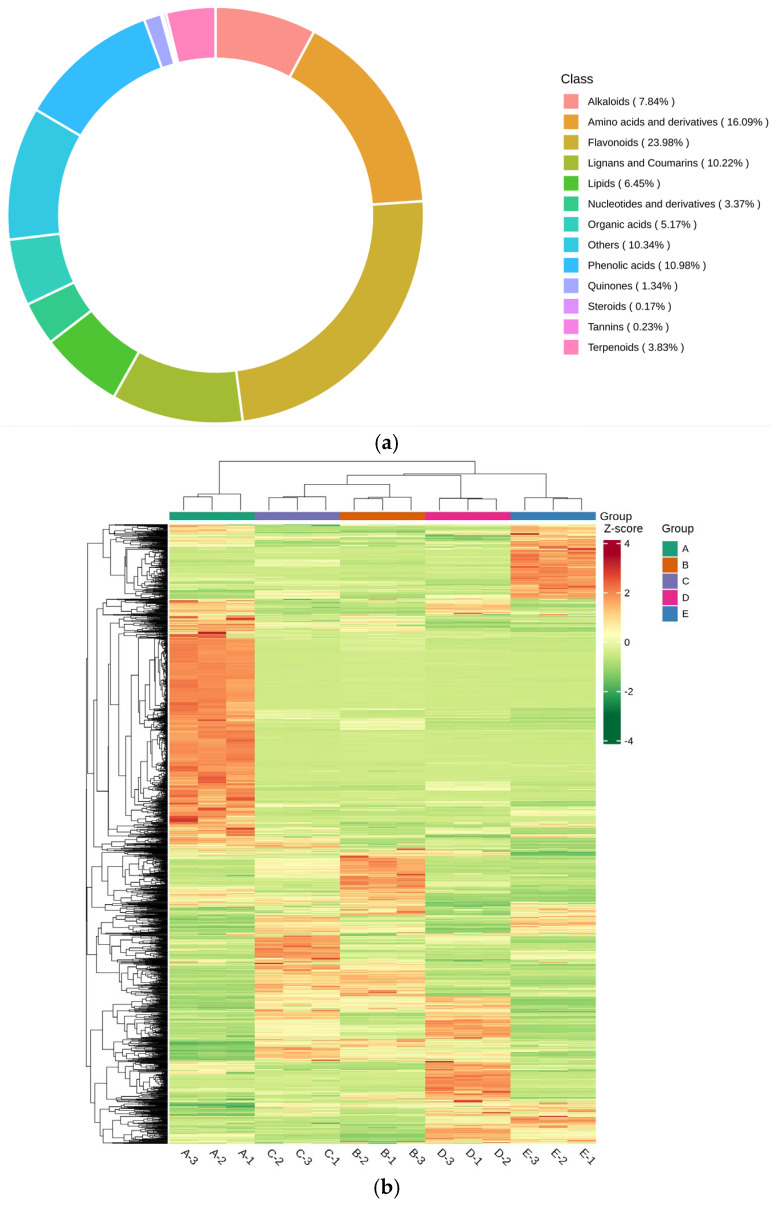
Metabolite category composition ring (**a**) and clustering heatmap (**b**) of five parts of the Shatian pomelo.

Based on the UPLC-MS/MS detection platform and the self-constructed database, a total of 1722 metabolites were detected in the samples, including 413 flavonoids, 89 organic acids and their derivatives, 277 amino acids and their derivatives, 189 phenolic acids, 111 lipids, 58 nucleotides and their derivatives, 135 alkaloids, 66 terpenoids, 23 quinones, 4 tannins, 3 steroids, 176 species of lignans and coumarins, and 178 species of other classes (Figure 3a). The other categories included 75 sugars, 10 alcohols, 19 vitamins, 11 ketones, 4 aldehydes, 12 lactones, and others. The results showed that flavonoids and amino acids and their derivatives had the highest relative content of 23.98% and 16.09%, respectively. The accumulation of metabolites in clustered heat maps showed significant differences in the pattern of metabolite abundance in different tissues (Figure 3b). The different parts of the Shatin pomelo were clustered into four categories: **A** (exocarp) was the first category and was the most different from the other three categories, **B** (exocarp) and **C** (segment membrane) were the second, **D** (pulp) was the third, and **E** (seeds) was the fourth category. This indicated that the metabolite differences between endocarp and segment membrane are minimal.

### 3.3. Differential Screening of Metabolites from Different Parts of the Shatian Pomelo

Orthogonal Partial Least Squares Discriminant Analysis (OPLS-DA) is a multivariate statistical analysis method for supervised pattern recognition, which is also effective in extracting information for variables with small correlations. Compared with PCA, PLS-DA can maximize the distinction between groups and facilitate the discovery of differential metabolites. Orthogonal Partial Least Squares Discriminant Analysis (OPLS-DA) combines Orthogonal Signal Correction (OSC) and PLS-DA methods, and is able to decompose X-matrix information into two categories of information related to Y and irrelevant information, and screen for differential variables by removing irrelevant differences. In OPLS-DA, the raw data are log2 transformed and then centered, where X is the sample quantitative information matrix and Y is the sample grouping information matrix.

According to the results of the OPLS-DA score plot (Figure S2), the score arrangement between different parts of the Shatian pomelo appeared in pairs on both sides of the distribution, indicating that there were significant differences in the metabolites between the two parts. In the OPLS-DA model validation, R^2^X and R^2^Y indicate the explanation rate of the model on the X and Y matrices, respectively, and Q^2^ indicates the predictive ability of the model. The closer the three indexes are to 1, the more stable and reliable the model; a model can be regarded as a valid model when Q^2^ > 0.5, a better model when Q^2^ > 0.9, and in general, *p* < 0.05 for Q^2^ indicates the best model. The R^2^Y and Q^2^ scores of the two-by-two comparison of OPLS-DA model validation for different parts of the Shatian pomelo were all above 0.9 and *p* < 0.005 for all Q^2^s, indicating that the model is very suitable (Figure S3).

**Figure 4:**
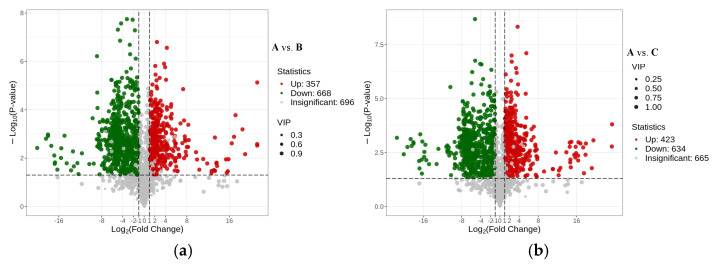

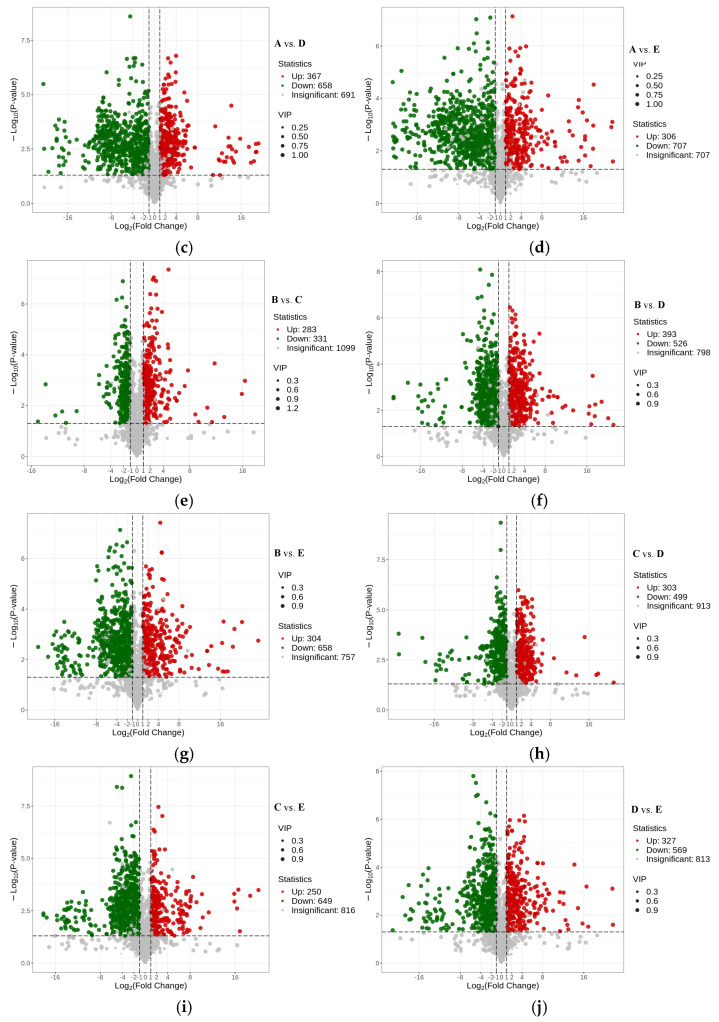
Volcano plots of differential metabolites between different parts (**A**—exocarp, **B**—endocarp, **C**—segment membrane, **D**—pulp, and **E**—seed) of the Shatian pomelo. ((**a**) **A** vs. **B**, (**b**) **A** vs. **C**, (**c**) **A** vs. **D**, (**d**) **A** vs. **E**, (**e**) **B** vs. **C**, (**f**) **B** vs. **D**, (**g**) **B** vs. **E**, (**h**) **C** vs. **D**, (**i**) **C** vs. **E**, (**j**) **D** vs. **E**).

**Figure 5:**
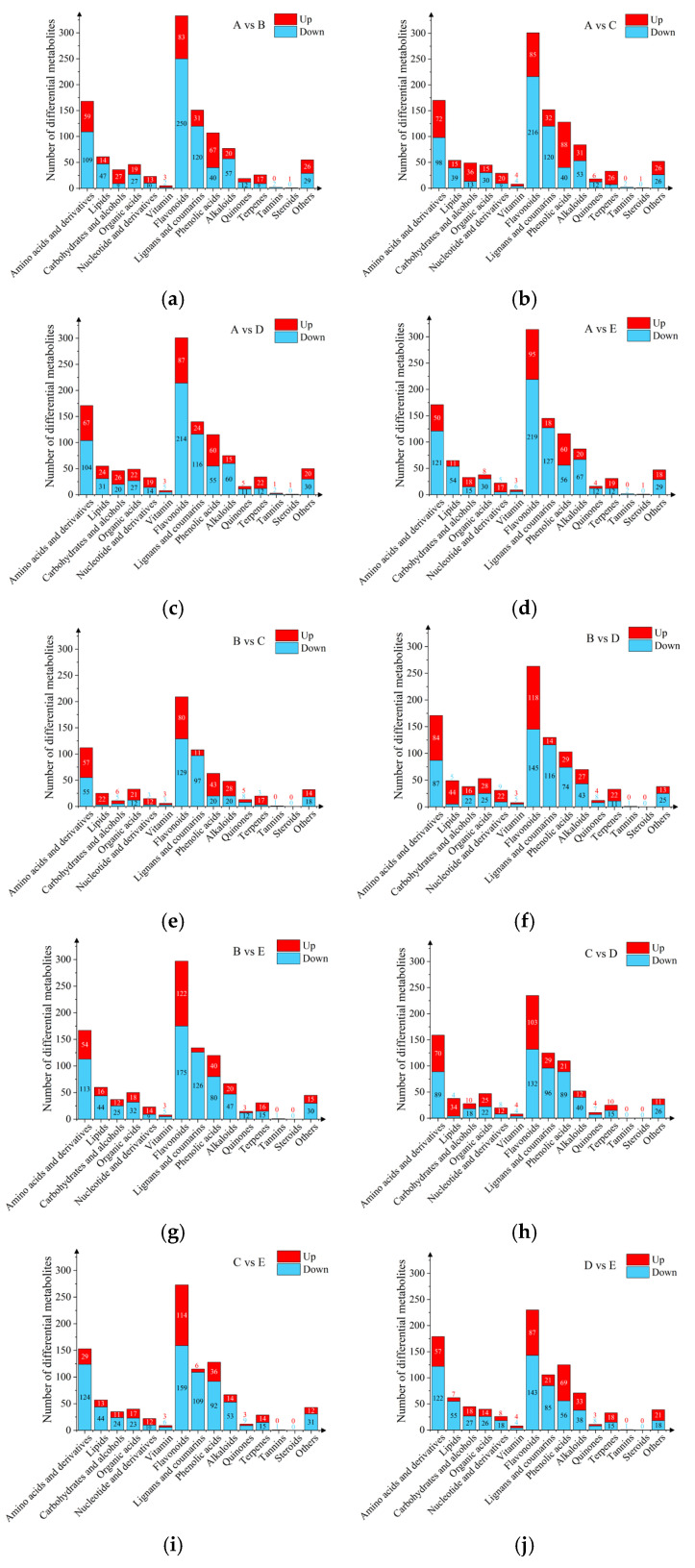
Types and quantity of differential metabolites between different parts (**A**—exocarp, **B**—endocarp, **C**—segment membrane, **D**—pulp, and **E**—seed) of the Shatian pomelo. ((**a**) **A** vs. **B**, (**b**) **A** vs. **C**, (**c**) **A** vs. **D**, (**d**) **A** vs. **E**, (**e**) **B** vs. **C**, (**f**) **B** vs. **D**, (**g**) **B** vs. **E**, (**h**) **C** vs. **D**, (**i**) **C** vs. **E**, (**j**) **D** vs. **E**).

Based on the OPLS-DA model results of Variable Importance in Projection (VIP) VIP ≥ 1 and Fold Change in Difference (FC) ≥ 2 or ≤ 0.5, the metabolites that differed among different parts of Shatian pomelo were screened out (Figure 4). The analysis of different parts of the Shatian pomelo showed that there were significant differences in metabolites among different parts. Pairwise comparisons of metabolites in each group of **A**, **B**, **C**, **D**, and **E** (**A** vs. **B**, **A** vs. **C**, **A** vs. **D**, **A** vs. **E**, **B** vs. **C**, **B** vs. **D**, **B** vs. **E**, **C** vs. **D**, **C** vs. **E**, and **D** vs. **E**) screened out 1110, 1126, 1097, 1097, 696, 1000, 1055, 895, 984, and 976 differential metabolites, respectively (Figure 5a–j). The exocarp (**A**) of the Shatian pomelo had the most differential metabolites from other tissue (Figure 3b). In addition, there were relatively few differential metabolites between the endocarp (**B**) and the segment membrane (**C**), and a total of 696 metabolites were screened, of which 377 metabolites showed downregulation and 319 metabolites showed upregulation. Flavonoids, amino acids and their derivatives, lignans, coumarins, phenolic acids, and alkaloids were the primary differential metabolites, accounting for more than 70% of the total differential metabolites.

### 3.4. Annotation and Enrichment Analysis of KEGG for Differential Metabolites from Different Parts of the Shatian Pomelo

**Figure 6:**
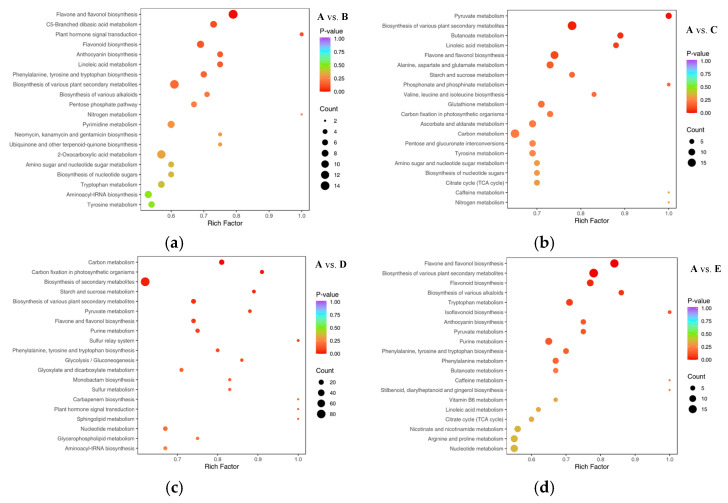

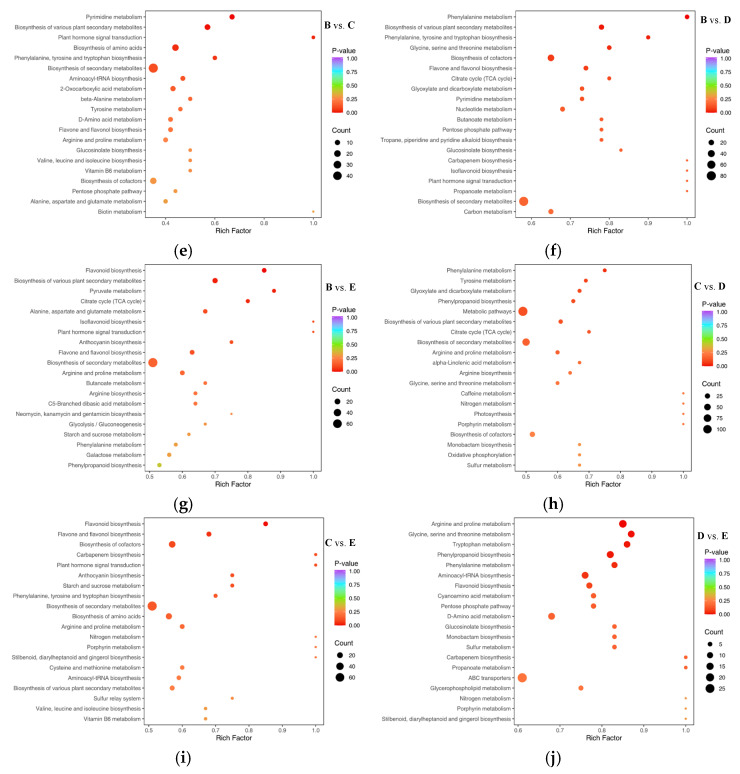
KEGG enrichment of differential metabolites between different parts of the Shatian pomelo ((**a**) **A** vs. **B**, (**b**) **A** vs. **C**, (**c**) **A** vs. **D**, (**d**) **A** vs. **E**, (**e**) **B** vs. **C**, (**f**) **B** vs. **D**, (**g**) **B** vs. **E**, (**h**) **C** vs. **D**, (**i**) **C** vs. **E**, (**j**) **D** vs. **E**).

KEGG pathway enrichment analysis was performed based on the results of differential metabolites, where the Rich Factor is the ratio of the number of differential metabolites in the corresponding pathway to the total number of metabolites annotated to that pathway, and a larger value indicates a greater degree of enrichment. Each bubble in Figure 6 represents a metabolic pathway, and the larger the bubble size, the greater the number of differentially significant metabolites enriched into the corresponding pathway. The color of the bubbles represents the *p*-value of the enrichment analysis, and the darker the color, the more significant the degree of enrichment; the top 20 pathways ranked by *p*-value were selected in the figure. Through the statistics of KEGG pathways of differential metabolites, it was found that the differential metabolites of **A** vs. **B** were enriched in 93 pathways (Figure 6a). The main enriched metabolic pathways were the synthesis of flavones and flavonols, and C5-branched dibasic acid metabolism. The differential metabolites of **A** vs. **C** were enriched in 95 pathways (Figure 6b), among which the main enriched metabolic pathways were the metabolism of pyruvate and the biosynthesis of various secondary metabolites in plants. Differential metabolites of **A** vs. **D** were enriched in 98 pathways (Figure 6c), in which the main enriched metabolic pathways were carbon metabolism, carbon fixation by photosynthetic organisms, and biosynthesis of secondary metabolites; and differential metabolites of **A** vs. **E** were enriched in 90 pathways (Figure 6d), in which the main enriched metabolic pathways were the synthesis of flavonoids and flavonols, the biosynthesis of various secondary metabolites in plants, and synthesis of flavonoids. In addition, the metabolic pathway of different parts of the Shatian pomelo is mainly biosynthesis of various secondary plant metabolites, including flavonols, flavonoids, anthocyanins, isoflavones, phenylalanines, alkaloids, lignin, and other biosynthesis. The above results indicated that the metabolic pathways of flavonoid metabolites and amino acids and their derivatives are the most prominent KEGG pathways among the differential metabolites.

### 3.5. Key Differential Metabolites from Different Parts of Shatian Pomelo

**Figure 7:**
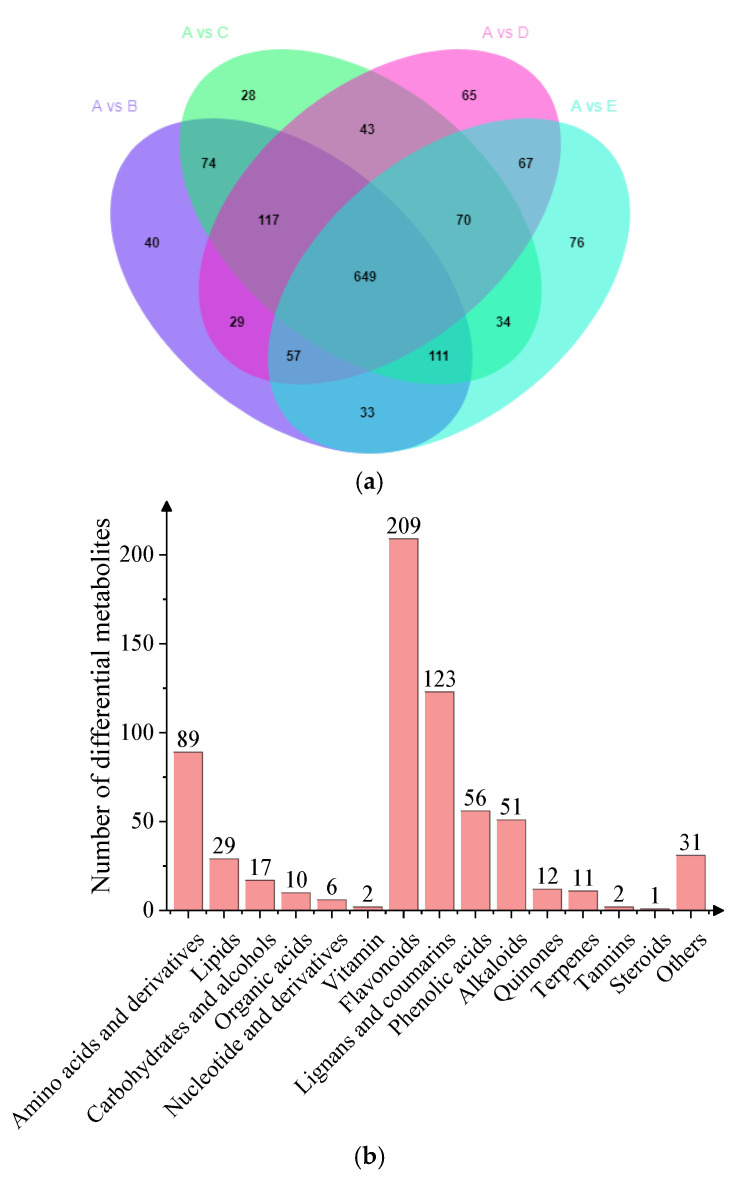
Wayne diagrams of differential metabolites (**a**) and key differential metabolite types and quantity in different parts of the Shatian pomelo (**b**) (**A** vs. **B**, **A** vs. **C**, **A** vs. **D**, **A** vs. **E**).

As shown in Figure 7, the exocarp (**A**) of the Shatian pomelo was significantly different from other tissue parts in terms of composition. The exocarp (**A**) was used as a control to analyze and compare the key differential metabolites of different parts of the Shatian pomelo. The results showed that a total of 649 key differential metabolites overlapped in **A** vs. **B**, **A** vs. **C**, **A** vs. **D**, and **A** vs. **E**, most of which were downregulated in expression. Among them, there were 209 flavonoid differential metabolites, accounting for 32.20%; 123 lignans and coumarins differential metabolites, accounting for 18.95%; 89 amino acids and their derivatives differential metabolites, accounting for 13.71%; 56 phenolic acids differential metabolites, accounting for 8.63%; and 51 alkaloids differential metabolites, accounting for 7.86%.

**Figure 8:**
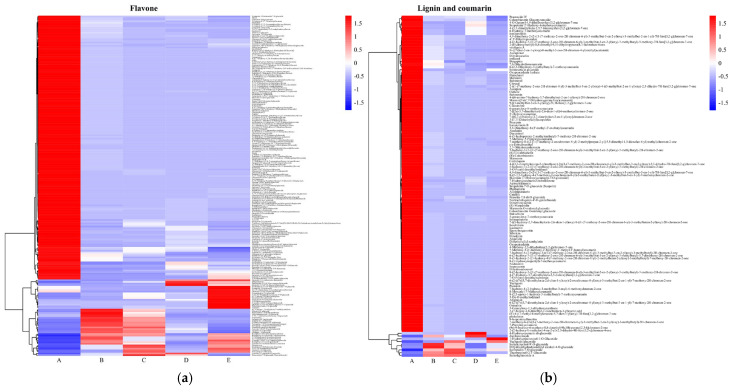

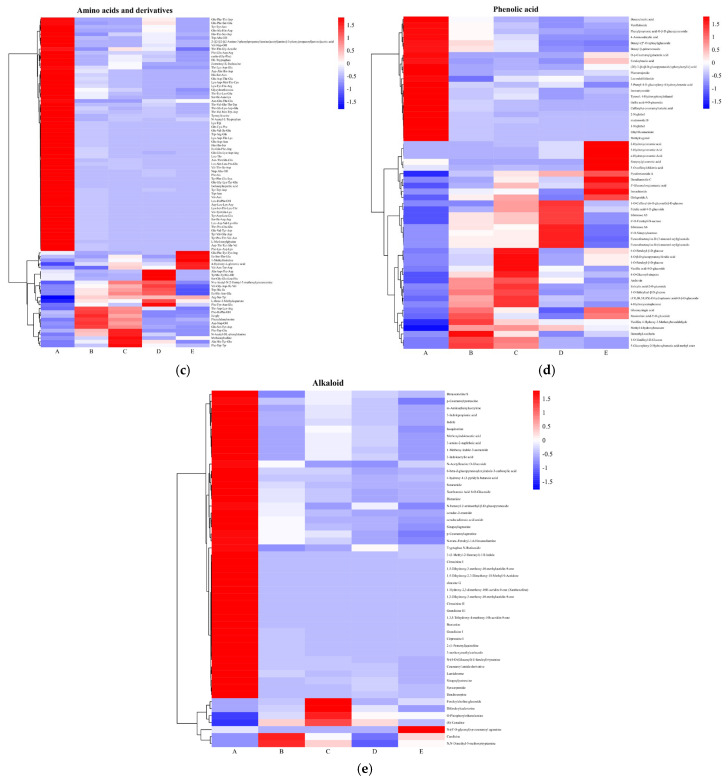
Heat map of clustering of major key differential metabolites in different parts of the Shatian pomelo ((**a**) flavonoid, (**b**) lignin and coumarin, (**c**) amino acids and their derivatives, (**d**) phenolic acid, (**e**) alkaloids, (**f**) lipids).

The clustering heat map of the main key differential metabolites from different parts of Shatian pomelo is shown in Figure 8. Among the 209 flavonoid differential metabolites screened, 162 flavonoid metabolites were specific to the exocarp (**A**), including 105 flavonoids, 32 flavonols, seven isoflavonoids, three chalcones, five dihydroflavonoids, one dihydroflavonol, five anthocyanins, and four anthrone. The 10 most abundant flavonoids were 5,4′-dihydroxy-3,6,7,3′-tetramethoxyflavone, 5,7-dihydroxy-6,3′,4′,5′-tetramethoxyflavone, 5,7-dihydroxy-3,4′,6,8-tetramethoxyflavone, 5,7,4′-trihydroxy-8,3′-dimethoxyflavone, 3,3′,4′,5′-tetrahydroxy-5,7-dimethoxyflavone, 3,3′,5,7-tetrahydroxy-4′,6-dimethoxyflavone, diosmetin-7-O-rutinoside, peonidin-3-O-(6′′-O-p-coumaroyl) glucoside, cathelicidin F, and skullcapflavone II (Table 1). In addition, 20 flavonoid metabolites were not detected in the seeds (**E**). Among them, Chrysoeriol-5-O-glucoside and Limocitrol 3-Glucoside were detected only in the exocarp (**A**) and the endocarp (**B**). Chrysoeriol-7-O-sophoroside-5-O-glucuronide was detected only in the exocarp (**A**) and the segment membrane (**C**), with a significantly higher relative content in the exocarp compared to the segment membrane.

Among the 123 lignan and coumarin-like differential metabolites screened, 114 metabolites were specific for the exocarp (**A**), including 100 coumarins and four lignans. The 10 most abundant lignans and coumarins were Meranzin, Suberenol, Citrusal, Isopeucenin, Peucenin, 4,9-dimethoxy-2-(2-((1-(7-methoxy-2-oxo-2H-chromen-6-yl)-3-methylbut-3-en-2-yl)oxy)-3-methylbut-3-en-1-yl)-7H-furo[3,2-g]chromen-7-one, Marmesin, (R)-Columbianetin, (S)-Columbianetin, and Aculeatin (Table 1). Among the 89 amino acids and their derivatives screened for differential metabolites, 63 metabolites were specific for exocarp (**A**), of which the 10 most abundant amino acids and their derivatives were histidine-histidine-serine (His-His-Ser), Nap-Abu-OH, Thr-Thr-Gly-Leu-Ile, glutamic acid-aspartic acid- Threonine-Glutamic acid (Glu-Asp-Thr-Glu), L-Methionylglycine, Phe-Lys-Asp-Lys, N-Acetyl-L-Tryptophan, Lys-Asp-Met-Thr-Cys, Glycylmethionine, and Tryptophan-Asparagine (Trp-Asn).

Among the 56 phenolic acid differential metabolites identified, 21 metabolites were specific to the exocarp (**A**). Of these, the 10 most abundant phenolic acid metabolites include vanillol glycoside (Vanilloloside), ethylrosmarinate, isomartynoside, O-coumaroylgalactaric acid, caffeoyl-p-coumaroyltartaric acid, gallic acid-4-O-glucoside, benzyl β-primeveroside, phenylpropionic acid-O-β-D-glucopy ranoside, benzyl-(2″-O-xylosyl) glucoside, and mudanoside B (Table 1).

Among the 51 alkaloid differential metabolites screened, 44 metabolites were specific to the exocarp (**A**), including 20 alkaloids, eight phenolic amines, eight indole alkaloids, five quinoline alkaloids, one isoquinoline alkaloid, one pyridine alkaloid, and one pyrrole alkaloid. Among them, the 10 most abundant alkaloid metabolites were 1,2-Dihydroxy-3-methoxy-10-methylacridin-9-one, clausine G, 1-Hydroxy-2,3-dimet hoxy-10H-acridin-9-on, Grandisine III, p-Coumaroylputrescine, CitpressineI,4,8-dihydroxyquinoline8-O-glucoside, m-Aminophenylacetylene, 1,5-Dihydroxy-3- methoxy-10-methylacridin-9-one, and indole (Table 1). In addition, 1,5-dihydroxy-3-methoxy-10-methylacridin-9-one was not detected in the pulp (D), but was detected in the endocarp (**B**), segment membrane (**C**), and seeds (the content was lower in **E**), but the content wass highest in the exocarp (**A**).

## 4. Discussion and Analysis

Compared with other metabolomics methods, widely targeted metabolomics has a wide coverage and high sensitivity, and can comprehensively quantitatively and qualitatively analyze microbial, animal, and plant metabolites [18,19]. This technique allows comprehensive and systematic data to be obtained for the analysis of metabolites in various tissue parts of the Shatian pomelo. A total of 1722 metabolites were detected in this study (Table S1), including primary metabolites such as amino acids and their derivatives (16.09%), nucleotides and their derivatives (3.37%), sugars and alcohols (4.94%), and secondary metabolites such as flavonoids (23.98%), phenolic compounds (10.98%), and alkaloids (7.84%). Plant components have different metabolic forms in different organs [20]. From the principal component analysis plot in Figure 3, it was concluded that there was a significant difference between the components of the exocarp (A) and the other parts of the plant, which is in agreement with what Wang et al. [21] found in their metabolic study of citrus fruits. Secondly, there was also a significant difference between the metabolites of seeds (E) and other parts, which may be related to the various metabolic activities of pomelo fruits during the growth and development process [22,23,24]. From Figure 8, it was concluded that there were significant distribution differences in the content of metabolites in different parts of the Shatian pomelo. The exocarp (A) contained a total of 682 high-content metabolites, mainly flavonoids (Chrysosplenetin, Arteanoflavone, 5,7-Dihydroxy-3,4’,6,8-tetramethoxyflavone, etc.), lignans and coumarins (Meranzin, Suberenol, Citrusal, etc.), and amino acids and their derivatives (L-Tryptophan, His-His-Ser, Nap-Abu-OH, etc.). The endocarp (B) contained a total of 229 high-content metabolites, mainly flavonoids (Naringenin-7-O-(6″-malonyl)glucoside, 3’,5,5’,7-Tetrahydroxyflavanone-7-O-glucoside, Dihydrokaempferol-3-O-glucoside, etc.), amino acids and their derivatives (N(6),N(6)-Dimethyl-L-lysine, Arg-Ser-Tyr, L-Arginine etc.), and phenolic acids (Phloroglucinol-1-O-β-D-glucopyranoside, Dimethyl phthalate, Protocatechuic acid-4-O-glucoside, etc.). The segment membrane (C) contained a total of 233 high-content metabolites, mainly flavonoids (4’-O-Glucosylvitexin, Vitexin-2″-O-galactoside, Apigenin-5-O-glucoside, etc.), amino acids and their derivatives (Phe-Trp-Tyr, L-Glutamine, L-Lysine, etc.), and phenolic acids (Phloroglucinol-1-O-β-D-glucopyranoside, 4-O-β-D-glucopyranosylferulic acid, 1-O-Feruloyl-β-D-glucose, etc.). The pulp (D) contained 297 high-abundance metabolites, and the primary components were amino acids and their derivatives (L-Aspartic Acid, N (6), N (6)-Dimethyl-L-lysine, L-Glutamic acid, etc.), flavonoids (Cynaroside, Baohuoside I, Tamarixin, etc.), and lipids (LysoPC 16:0(2n isomer), LysoPC 16:0, Stearic Acid, etc.). Seeds (E) contained a total of 285 high-content metabolites, the main components of which were flavonoids (Amoenin, Tamarixin, Cyanidin-3-O-galactoside, etc.), followed by phenolic acids (3-Hydroxycinnamic Acid, α-Hydroxycinnamic Acid, 2-Hydroxycinnamic acid, etc.), and amino acids and their derivatives (Tyr-Phe-Trp, Arg-Ser-Tyr, L-Lysine, etc.). Among them, D-Pinitol (D-Pinitol) and D-Xylonic acid (D-Xylonic acid) were close to each other in the endocarp (B) and segment membrane (C), Diosphenol 2-(6″-Malonyl) Glucoside was close in both the segment membrane (C) and seed (E), and Ellagic acid-4-O-xyloside (Ellagic acid-4-O-Xyloside) was close in the endocarp (B) and pulp (D). The largest variety of metabolites was detected in flavonoids, with a total of 413 species, accounting for 23.98% of the total metabolites; mainly flavonoids such as Chrysosplenetin (A > B > C > E > D) and amoenin (E > D > C > B > A), flavonols such as limocitrin (A > B > C > E > D), and anthocyanins such as Peonidin-3-O-(6″-O-p-coumaroyl)glucoside (A > D > E > C > B) [25,26]. Naringin (B > C > D > E > A) is the predominant flavonoid in various parts of the pomelo, including the exocarp, endocarp, segment membrane, pulp, and seeds. Neoeriocitrin is the predominant flavonoid component found in seeds [27]. Nobiletin (B > D > C > A > E), a plant-derived flavonoid, has been shown to have anti-inflammatory, anti-diabetic, and antioxidant properties [28,29], and has also been found to have therapeutic effects on Alzheimer’s disease [30]. A large number of studies have shown that flavonoids have a variety of bioactive functions, such as antioxidant activity, anticancer activity, prevention of cardiovascular and cerebrovascular diseases, etc. [31]. The metabolites derived from amino acid derivatives ranked second in terms of variety, with a total of 277 detected, including L-Tryptophan (A > C > B > E > B), Nap-Abu-OH (A > D > B > C > E), and Thr-Thr-Gly-Leu-Ile (A > C > D > B > E), etc. In addition, many citrullinated compounds have the potential to be developed as plant-derived drugs for the prevention and treatment of human diseases [32]. Analysis of KEGG pathway enrichment based on the differential metabolite results indicated that the metabolic pathway of flavonoid metabolites and the metabolic pathway of amino acids and their derivatives were the predominant differential metabolite KEGG pathways in pomelo fruit. This is consistent with the finding of flavonoid metabolites and amino acid metabolites as the major differential metabolite types in the assay results. This finding also suggests that the biosynthesis and metabolism of amino acids and flavonoids may be regulated in a tissue-specific manner, thus presenting a tissue-specific accumulation of amino acids and flavonoids in the pomelo [33]. This is consistent with previously reported findings on metabolite accumulation in citrus fruits. The exocarp had the highest content and variety of amino acids and their derivatives compared to other tissue sites, which may be due to the fact that amino acids are important primary metabolites and precursors for the synthesis of a variety of secondary metabolites, such as flavonoids, which accumulate through metabolic pathways, thus presenting a tissue-specific accumulation of metabolite content.

## 5. Conclusions

In this study, metabolites in Shatian pomelo fruits were systematically studied by means of widely targeted metabolomics technology, and the distribution of compound composition in different parts of the Shatian pomelo was clarified, which confirmed that widely targeted technology is a feasible scheme to study the quality characteristics of the pomelo. The results showed that the accumulation of various metabolites in the pomelo presented obvious tissue specificity, among which the metabolic pathways of flavonoid metabolites and amino acids and their derivatives were the most dominant differential metabolite KEGG pathways in pomelo fruits. This study lays a scientific foundation for metabolic research on the Shatian pomelo and citrus fruits. Key discoveries of flavonoids and amino acids offer promising avenues for functional foods, supplements, and therapies targeting chronic diseases. Insights into metabolic pathways underpinning the Shatian pomelo’s health benefits pave the way for targeted nutraceutical and pharmaceutical innovations.

## Figures and Tables

**Table 1:** The top 10 differential metabolites of flavonoids, lignans and coumarins, phenolic acids, and alkaloids with the highest abundance in the five parts of the Shatian pomelo.

NO.	RT (min)	Compounds	A (Exocarp)	B (Endocarp)	C (Segment Membrane)	D (Pulp)	E (Seed)
1	7.17	Chrysosplenetin (5,4′-Dihydroxy-3,6,7,3′-tetramethoxyflavone)	215,375,441.33 ± 2,733,016.92 ^a^	3,649,223.62 ± 255,425.12 ^b^	1,038,869.23 ± 369,062.40 ^c^	151,693.63 ± 21,734.25 ^c^	17,849.01 ± 5711.43 ^c^
2	7.01	5,7-Dihydroxy-6,3’,4’,5’-tetramethoxyflavone (Arteanoflavone)	199,144,747.6 ± 7,509,612.73 ^a^	3,257,368.39 ± 23,451.55 ^b^	1,058,011.09 ± 63,027 ^b^	141,366.86 ± 21,684.15 ^b^	14,512.12 ± 8195.76 ^b^
3	7.07	5,7-Dihydroxy-3,4’,6,8-tetramethoxyflavone	197,634,943.6 ± 2,221,127.57 ^a^	3,537,275.17 ± 104,667.83 ^b^	1,077,954.74 ± 96,963.36 ^c^	161,430.72 ± 8528.39 ^c^	18,517.77 ± 7373.69 ^c^
4	5.91	Limocitrin (5,7,4’-trihydroxy-8,3’-dimethoxyflavone)	115,680,954.87 ± 3,355,958.14 ^a^	4,032,390.53 ± 402,918.75 ^b^	870,832.43 ± 43,265.91 ^c^	262,394.37 ± 234,964.59 ^c^	49,824.69 ± 27,280.1 ^c^
5	5.90	3,3’,4’,5′-Tetrahydroxy-5,7-Dimethoxyflavone	112,800,123.7 ± 2,069,998.79 ^a^	4,015,686.73 ± 434,379.65 ^b^	883,382.07 ± 73,676.55 ^c^	287,122.36 ± 194,795.13 ^c^	27,163.77 ± 3921.35 ^c^
6	5.97	3,3’,5,7-Tetrahydroxy-4’,6-Dimethoxyflavone; (Laciniatin)	110,438,621.63 ± 4,248,654.88 ^a^	4,074,597.52 ± 899,302.78 ^b^	925,358.73 ± 98,083.31 b^c^	304,048.92 ± 174,264.63 ^d^	59,913.79 ± 27,814.35 ^d^
7	4.21	Diosmetin-7-O-rutinoside (Diosmin)	92,297,944.63 ± 8,557,177.77 ^a^	662,131.08 ± 53,129.53 ^c^	1,049,026.34 ± 246,757.27 ^c^	10,172,673.97 ± 1,612,973.6 ^b^	2,680,246.94 ± 511,396.29 ^c^
8	4.20	Peonidin-3-O-(6″-O-p-coumaroyl) glucoside	84,259,605.36 ± 13,964,244.65 ^a^	482,497.71 ± 44,279.46 ^b^	885,215.97 ± 51,186.07 ^b^	9,159,306.23 ± 287,503.52 ^b^	2,395,267.42 ± 320,379.65 ^b^
9	6.77	Chrysosplenol F	76,432,865.01 ± 2,172,940.91 ^a^	2,265,029.23 ± 293,596.33 ^b^	674,248.26 ± 41,185.12 ^bc^	116,875.73 ± 57,252.36 ^c^	19,009.09 ± 4735.11 ^c^
10	7.06	Skullcapflavone II	68,755,045.76 ± 1,504,580.52 ^a^	1,152,061.97 ± 33,998.7 ^b^	411,526.91 ± 34,949.46 ^bc^	58,736.67 ± 57,885.38 ^bc^	13,462.01 ± 898.31 ^c^
11	4.92	Meranzin	260,937,082.23 ± 23,304,358.27 ^a^	20,341,832.83 ± 1,708,360.9 ^b^	6,243,958.5 ± 144,435.99 ^bc^	12,543,136.5 ± 504,700.46 ^bc^	212,607.19 ± 18,038.13 ^c^
12	4.36	Suberenol	186,823,251.23 ± 21,776,116.02 ^a^	19,099,824.82 ± 1,699,241.51 ^b^	5,772,893.04 ± 28,612.4 ^bc^	12,435,930.24 ± 85,479.08 ^bc^	176,742.38 ± 5857.66 ^c^
13	4.54	Citrusal	186,823,251.23 ± 21,776,116.02 ^a^	19,099,824.82 ± 1,699,241.51 ^b^	5,772,893.04 ± 28,612.4 ^bc^	12,435,930.24 ± 85,479.08 ^bc^	176,742.38 ± 5857.66 ^c^
14	5.83	Isopeucenin	157,477,138.8 ± 12,162,157.68 ^a^	5,642,660.59 ± 1,312,269.86 ^b^	1,234,122.08 ± 65,187.29 ^b^	270,187.16 ± 192,250.21 ^b^	40,838.55 ± 9569.31 ^b^
15	8.00	Peucenin	154,394,924.77 ± 7,692,801.82 ^a^	6,430,933.69 ± 389,918.41 ^b^	2,062,597.8 ± 114,085.53 b^c^	364,476.85 ± 106,253.91 ^c^	75,073.57 ± 8458.71 ^c^
16	9.34	4,9-dimethoxy-2-(2-((1-(7-methoxy-2-oxo-2H-chromen-6-yl)-3-methylbut-3-en-2-yl) oxy)-3-methylbut-3-en-1-yl)-7H-furo[3,2-g] chromen-7-one	123,031,095.1 ± 5,623,842.42 ^a^	8,623,836.7 ± 1,685,324.27 ^b^	3,410,740.97 ± 818,112.44 ^c^	324,912.94 ± 93,832.91 ^c^	56,452.08 ± 25,491.96 ^c^
17	5.12	Marmesin	94,794,297.06 ± 13,634,909.66 ^a^	5,892,271.63 ± 308,200.36 ^b^	936,469.33 ± 127,144.45 ^b^	199,081.51 ± 46,710.36 ^b^	24,964.74 ± 1101.51 ^b^
18	5.27	(R)-Columbianetin	89,507,016.21 ± 10,018,326.9 ^a^	5,543,506.33 ± 162,326.75 ^b^	876,848.22 ± 63,002.84 ^b^	232,068.1 ± 16,449.96 ^b^	27,053.88 ± 5665.46 ^b^
19	5.16	(S)-Columbianetin	89,030,986.87 ± 7,601,557.45 ^a^	5,273,407.35 ± 203,666.33 ^b^	901,251.66 ± 49,519.62 ^b^	233,312.31 ± 33,575.65 ^b^	33,981.72 ± 4086.74 ^b^
20	5.29	Aculeatin	87,874,086.71 ± 9,177,839.7 ^a^	4,085,031.99 ± 141,979.16 ^b^	839,461.13 ± 74,198.74 ^b^	436,005.05 ± 30,257.15 ^b^	32,599.26 ± 8838.45 ^b^
21	1.74	Vanilloloside	66,918,314.54 ± 2,200,006.22 ^a^	2,2407,728.96 ± 1,936,019.52 ^b^	9,630,821.78 ± 264,738.34 ^c^	1,047,743.08 ± 33,178.48 ^e^	4,662,786.36 ± 289,863.42 ^d^
22	7.80	Ethyl Rosmarinate	45,477,614.42 ± 740,079.47 ^a^	1,014,298.25 ± 82,285.65 ^b^	314,092.07 ± 51,137.3 ^c^	76,242.37 ± 33,474.65 ^c^	8580.57 ± 5381.47 ^c^
23	4.53	Isomartynoside	20,687,319.68 ± 744,402.91 ^a^	2,429,958.35 ± 196,037.56 ^c^	3,581,404.68 ± 429,813.82 ^b^	242,327.76 ± 159,646.43 ^e^	1,676,687.08 ± 66,604.67 ^d^
24	2.51	O-p-Coumaroylgalactaric acid	15,489,567.74 ± 255,510.67 ^a^	35,983.57 ± 17,989.23 ^c^	40,145.37 ± 5073.08 ^c^	23,262.85 ± 11,225.3 ^c^	5,584,619.27 ± 375,002.9 ^b^
25	3.85	Caffeoyl-p-coumaroyltartaric acid	3,074,723.57 ± 191,997.19 ^a^	353,981.19 ± 23,437.76 ^b^	133,901.71 ± 13,837.88 ^c^	30,755.38 ± 12,655.1 ^c^	64,523.6 ± 9132.67 ^c^
26	2.10	Gallic acid-4-O-glucoside	2,837,670.36 ± 110,318.44 ^a^	290,698.33 ± 24,060.2 ^b^	95,060.68 ± 8822.36 ^c^	7424.4 ± 2166.22 ^d^	32,141 ± 8079.06 ^cd^
27	3.03	Benzyl β-primeveroside	2,586,449.41 ± 71,897.56 ^a^	1,129,773.84 ± 61,411.06 ^b^	701,857.19 ± 40,702.72 ^c^	114,193.4 ± 4212.4 ^d^	54,383.58 ± 10,190.7 ^d^
28	2.72	Phenylpropionic acid-O-β-D-glucopyranoside	2,413,478.4 ± 233,932.76 ^a^	778,878.41 ± 144,084.99 ^b^	249,482.8 ± 34,050.35 ^c^	85,555.14 ± 23,928.44 ^c^	57,981.33 ± 13,160.53 ^c^
29	3.29	Benzyl-(2′′-O-xylosyl) glucoside	2,254,227.17 ± 187,358.5 ^a^	1,025,592.16 ± 92,579.99 ^b^	674,646.37 ± 54,996.98 ^c^	98,671.06 ± 14,700.62 ^d^	47,044.06 ± 9160 ^d^
30	2.19	mudanoside B	2,122,356.04 ± 84,289.31 ^a^	90,109.14 ± 7076.37 ^b^	63,744.08 ± 4854.33 ^bc^	6147.13 ± 378.22 ^c^	14,332.81 ± 3346.47 ^c^
31	6.25	1,2-Dihydroxy-3-methoxy-10-methylacridin-9-one	76,802,456.09 ± 6,747,989.98 ^a^	208,713.08 ± 59,287.97 ^b^	38,123.64 ± 9474.95 ^b^	16,620.93 ± 12,116.89 ^b^	8218.07 ± 3394.31 ^b^
32	6.42	clausine G	75,466,395.59 ± 5,404,771.87 ^a^	184,078.51 ± 95,857.1 ^b^	32,248.41 ± 4504.21 ^b^	18,512.72 ± 16,919.5 ^b^	7231.26 ± 4518.78 ^b^
33	6.27	1-Hydroxy-2,3-dimethoxy-10H-acridin-9-one (Xanthoxoline)	75,122,410.3 ± 5,559,738.77 ^a^	199,659.79 ± 112,539.04 ^b^	45,933.71 ± 9408.16 ^b^	17,618.92 ± 18,214.61 ^b^	5988.84 ± 3300.55 ^b^
34	5.50	Grandisine III	47,531,920.36 ± 4,206,918.74 ^a^	156,240.64 ± 56,592.05 ^b^	28,639.62 ± 12,252.91 ^b^	19,751.77 ± 10,476.99 ^b^	15,743.45 ± 11,923.24 ^b^
35	2.47	p-Coumaroylputrescine	23,396,172.51 ± 3,501,029.57 ^a^	5,114,109.93 ± 366,920.37 ^b^	7,609,952.68 ± 480,229.08 ^b^	5,116,393.88 ± 81,310.33 ^b^	666,890.53 ± 25,260.76 ^c^
36	6.84	Citpressine I	17,813,550.77 ± 1,192,572.48 ^a^	232,362.3 ± 170,007.8 ^b^	8981.16 ± 1709.46 ^b^	4681.46 ± 2056.72 ^b^	4618.94 ± 852.09 ^b^
37	2.13	Xanthurenic Acid 8-O-Glucoside	16,172,791.27 ± 1,337,176.61 ^a^	3,473,240.25 ± 73,470.84 ^b^	1,936,397.29 ± 85,652.95 ^c^	744,633.09 ± 77,972.89 ^d^	1,023,957.8 ± 76,356.01 ^cd^
38	2.33	m-Aminophenylacetylene	13,541,353.9 ± 820,841.54 ^a^	3,237,092.85 ± 63,039.95 ^d^	4,896,596.8 ± 412,573.04 ^b^	4,178,287.91 ± 108,391.81 ^c^	3,124,474.58 ± 41,155.84 ^d^
39	6.79	1,5-Dihydroxy-3-methoxy-10-methylacridin-9-one	13,253,993.39 ± 41,434.9 ^a^	28,782.17 ± 11,677.93 ^b^	10,252.21 ± 3008.51 ^b^	-	7652.66 ± 3405.02 ^b^
40	2.47	Indole	12,696,287.5 ± 938,357.84 ^a^	3,490,247.27 ± 215,867.14 ^cd^	4,817,418.95 ± 468,345.87 ^b^	4,237,510.34 ± 85,488.53 ^bc^	3,200,661.16 ± 136,909.63 ^d^

Results are expressed as mean ± standard deviation (n = 3); different letters in a row indicate significant differences at the 5% level.

## Data Availability

The original contributions presented in the study are included in the article/Supplementary Material, further inquiries can be directed to the corresponding author.

## Supplementary Materials

The following supporting information can be downloaded at: https://www.mdpi.com/article/10.3390/foods13223698/s1, Figure S1: TIC plots of mass spectrometry detection of QC samples (a: positive ion mode, b: negative ion mode) and their TIC overlaps (c: positive ion mode, d: negative ion mode), TIC plots of multi-peak detection of metabolites in MRM mode (e: positive ion mode, f: negative ion mode); Figure S2: Plot of two-by-two comparison of OPLS-DA scores of different parts of Shatian pomelo (a: **A** vs. **B**, b: **A** vs. **C**, c: **A** vs. **D**, d: **A** vs. **E**, e: **B** vs. **C**, f: **B** vs. **D**, g: **B** vs. **E**, h: **C** vs. **D**, i: **C** vs. **E**, j: **D** vs. **E**); Figure S3 The R^2^Y and Q^2^ scores of the two-by-two comparison of OPLS-DA model validation for different parts of Shatian pomelo (a: **A** vs. **B**, b: **A** vs. **C**, c: **A** vs. **D**, d: **A** vs. **E**, e: **B** vs. **C**, f: **B** vs. **D**, g: **B** vs. **E**, h: **C** vs. **D**, i: **C** vs. **E**, j: **D** vs. **E**); Table S1. All sample data.
