# Supplementary material for: Widely Targeted Metabolomics Reveal the Distribution of Metabolites in Shatian Pomelo Fruit
Source: Foods. 2024 Nov 20;13(22):3698. doi: 10.3390/foods13223698 (PMC11593426; doi:10.3390/foods13223698)

Figure S1 TIC plots of mass spectrometry detection of QC samples (a: positive ion mode, b: negative ion mode) and their TIC overlaps (c: positive ion mode, d: negative ion mode), TIC plots of multi-peak detection of metabolites in MRM mode (e: positive ion mode, f: negative ion mode)

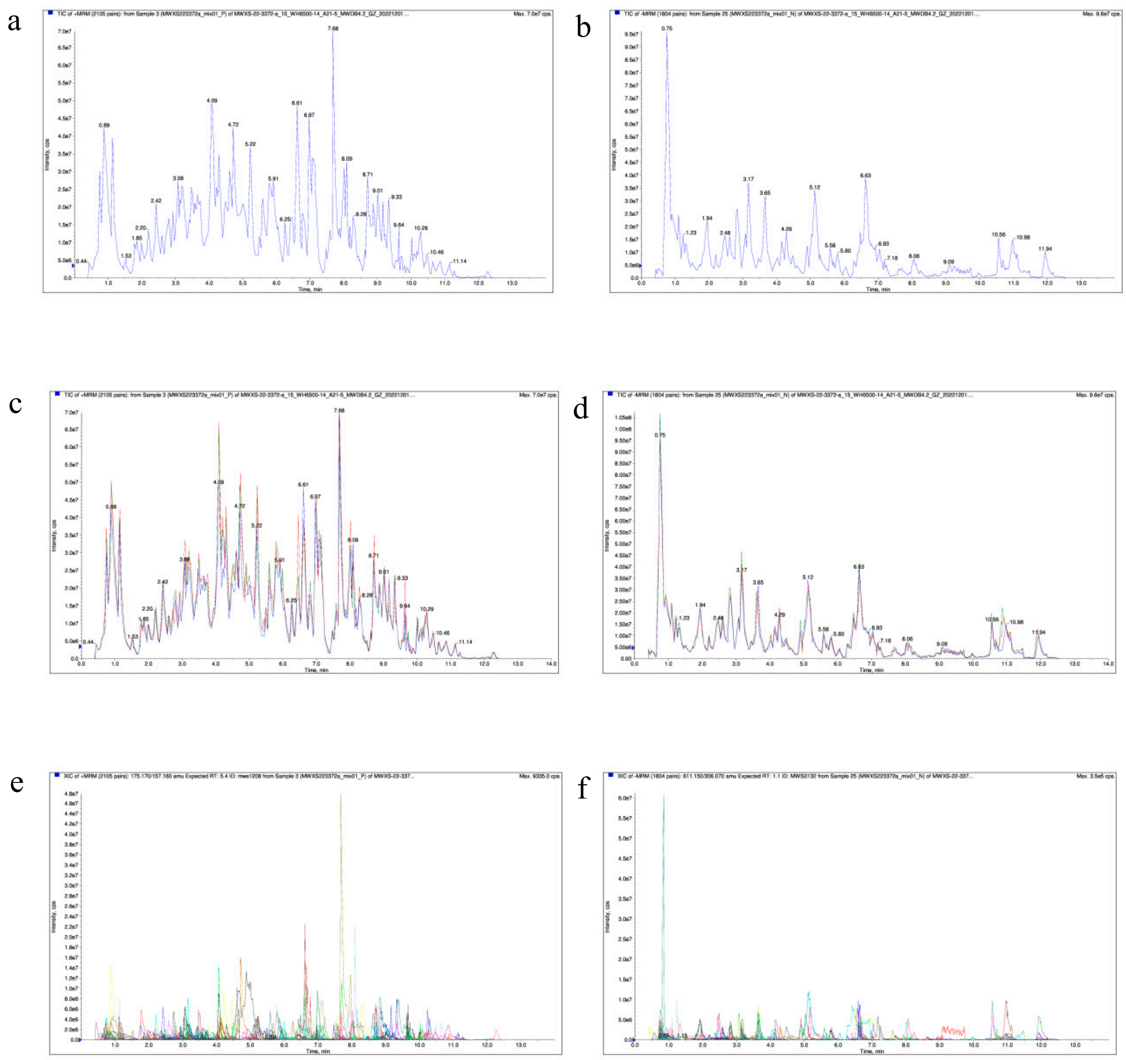

Figure S2 Plot of two-by-two comparison of OPLS-DA scores of different parts of Shatian pomelo (a: **A** vs **B**, b: **A** vs **C**, c: **A** vs **D**, d: **A** vs **E**, e: **B** vs **C**, f: **B** vs **D**, g: **B** vs **E**, h: **C** vs **D**, i: **C** vs **E**, j: **D** vs **E**)

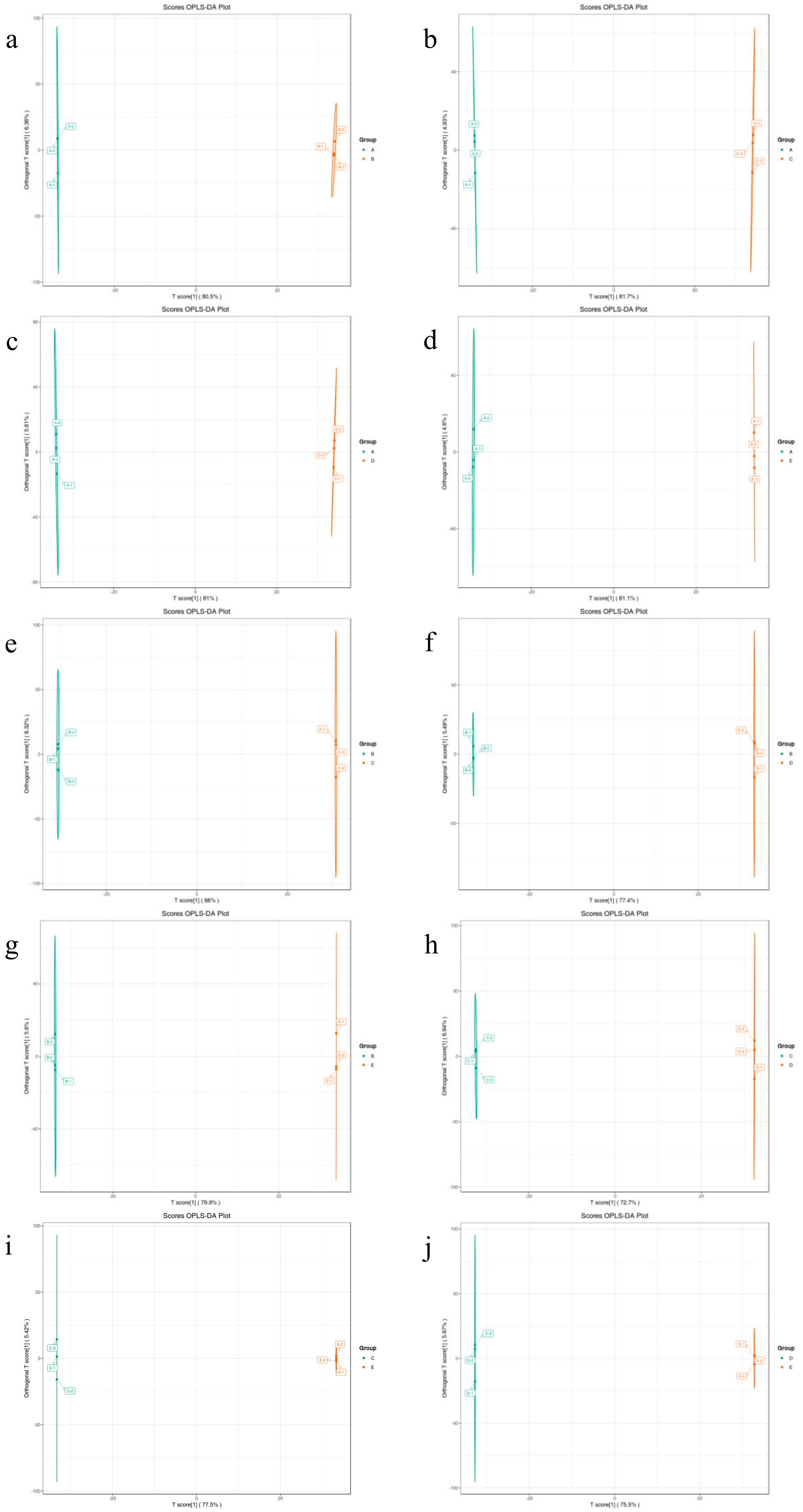

Figure S3 The R^2Y and Q^2 scores of the two-by-two comparison of OPLS-DA model validation for different parts of Shatian pomelo (a: **A** vs **B**, b: **A** vs **C**, c: **A** vs **D**, d: **A** vs **E**, e: **B** vs **C**, f: **B** vs **D**, g: **B** vs **E**, h: **C** vs **D**, i: **C** vs **E**, j: **D** vs **E**)

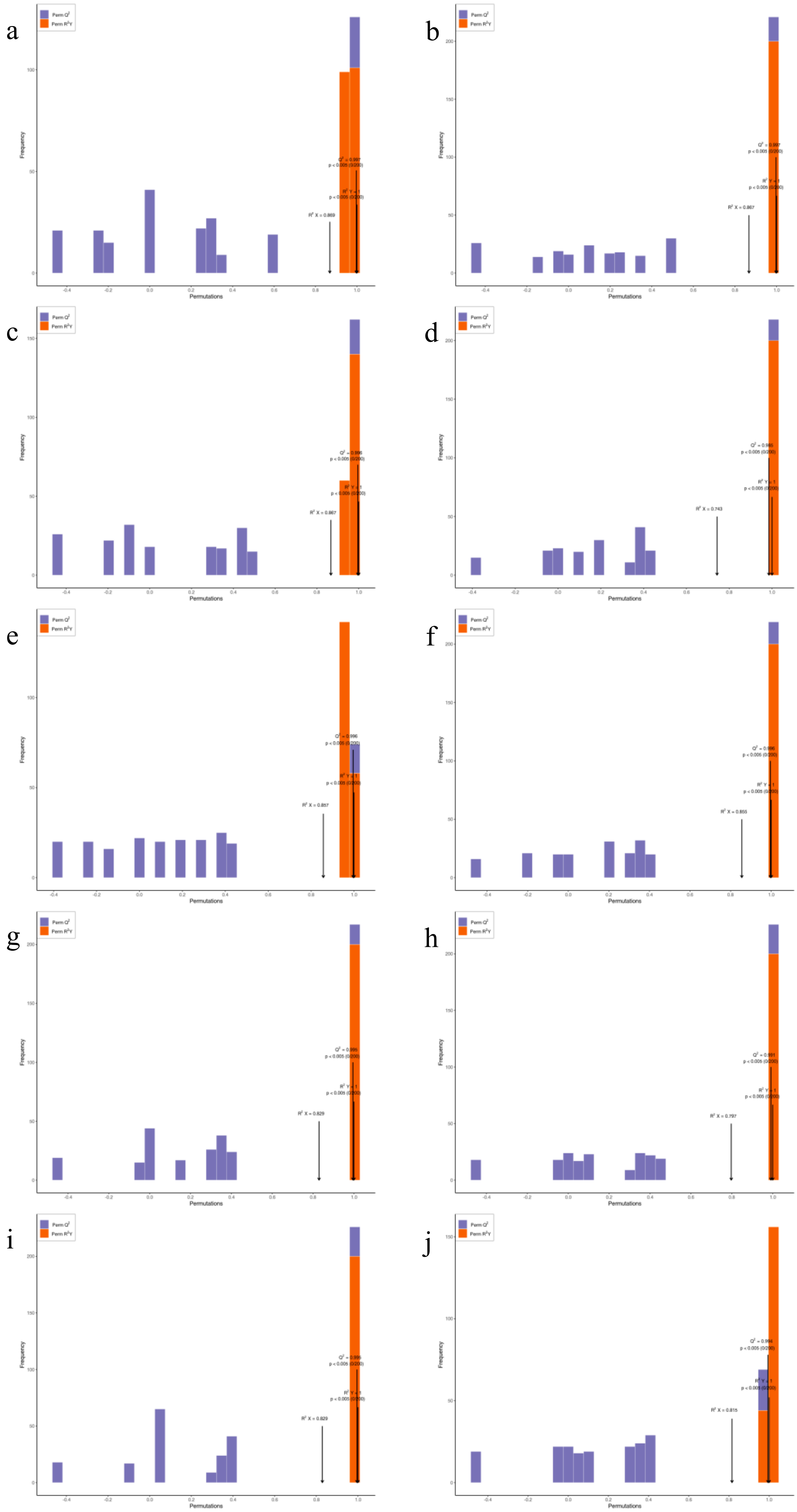

Supplement: Supplementary file 1 [file foods-13-03698-s001.zip › foods-3285166-supplementary.pdf]
